# Supplementary material for: Learning from fights: Males’ social dominance status impact reproductive success in Drosophila melanogaster
Source: PLoS One. 2024 Mar 7;19(3):e0299839. doi: 10.1371/journal.pone.0299839 (PMC10919672; doi:10.1371/journal.pone.0299839)
Supplement: S1 Fig — (A) The CVI extinction do not differ between Winners, Losers, Naïve 10min (Chi2 = 0.4 and p = 0.83) and (B) 60min (Chi2 = 3.5 and p = 0.17) after fight in non-competitive context. (C) However, CVI does decline faster in losers in competitive context against winners. (PDF) [file pone.0299839.s001.pdf]

S1 Figure

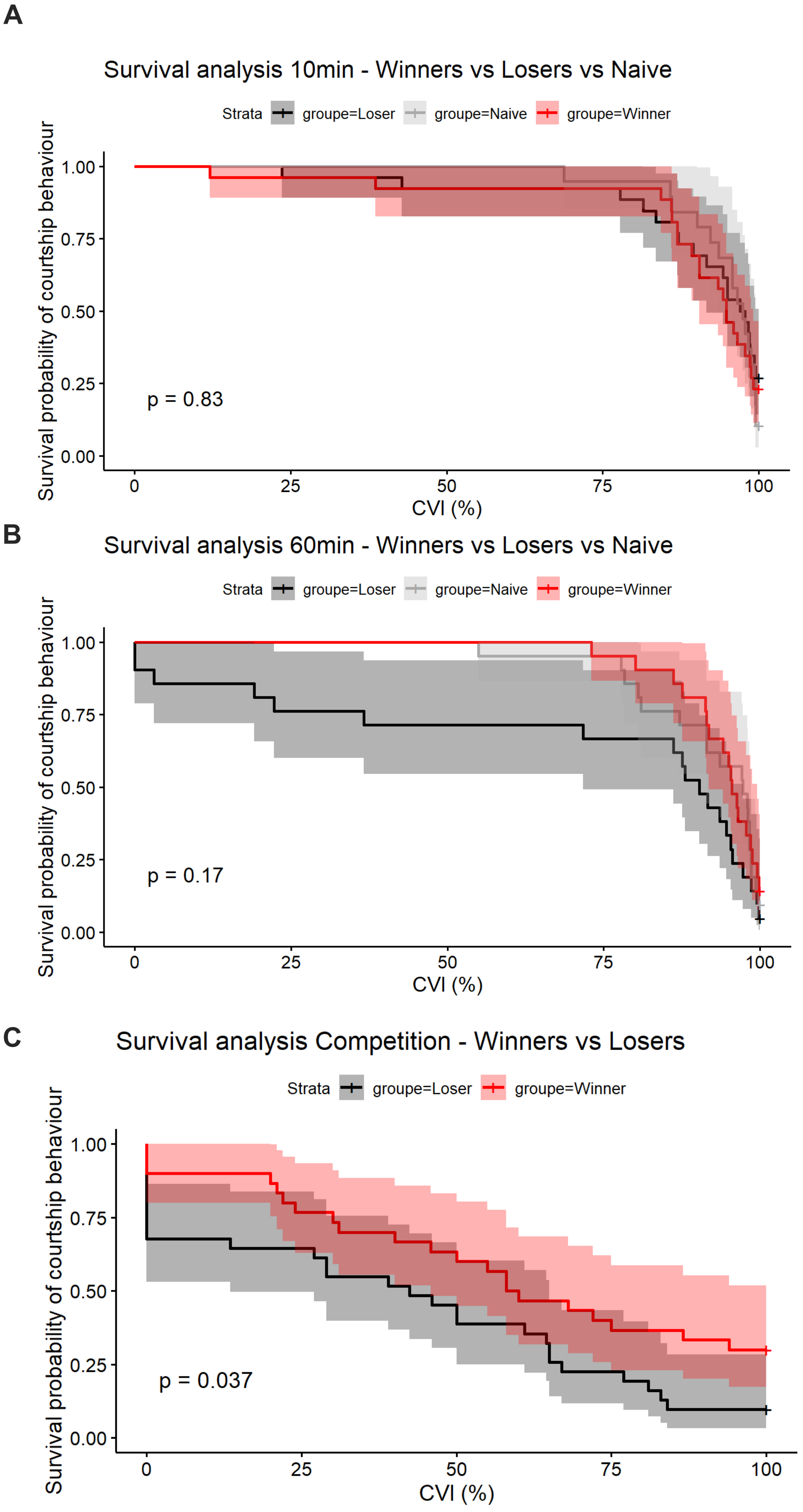

A
Survival analysis 10min - Winners vs Losers vs Naive
Strata groupe=Loser groupe=Naive groupe=Winner
Survival probability of courtship behaviour
1.00
0.75
0.50
0.25
0.00
p = 0.83
0 25 50 75 100
CVI (%)
B
Survival analysis 60min - Winners vs Losers vs Naive
Strata groupe=Loser groupe=Naive groupe=Winner
Survival probability of courtship behaviour
1.00
0.75
0.50
0.25
0.00
p = 0.17
0 25 50 75 100
CVI (%)
C
Survival analysis Competition - Winners vs Losers
Strata groupe=Loser groupe=Winner
Survival probability of courtship behaviour
1.00
0.75
0.50
0.25
0.00
p = 0.037
0 25 50 75 100
CVI (%)
